# ErbB2/HER2-specific NK cells for adoptive cancer immunotherapy

**DOI:** 10.1186/2051-1426-1-S1-P38

**Published:** 2013-11-07

**Authors:** Congcong Zhang, Michael Burger, Kurt Schönfeld, Sabrina Genβler, Christiane Sahm, Sonja Naundorf, Marcus Odendahl, Paulina Nowakowska, Torsten Tonn, Manuel Grez, Joachim P Steinbach, Winfried S Wels

**Affiliations:** 1 Georg-Speyer-Haus, Frankfurt, Germany; 2 Institute for Neurooncology, University Hospital, University of Frankfurt, Frankfurt, Germany; 3 EUFETS GmbH, Idar-Oberstein, Germany; 4 German Red Cross Blood Service North-East, Dresden, Germany; 5 German Red Cross Blood Service Baden-Württemberg - Hessen, Frankfurt, Germany

## 

Significant progress has been made over the last decade towards realizing the potential of natural killer (NK) cells for cancer immunotherapy. NK cells can respond rapidly to transformed and stressed cells, and have the intrinsic potential to extravasate and reach their targets in almost all body tissues. In addition to donor-derived primary NK cells, also continuously expanding cytotoxic cell lines such as NK-92 are being considered for adoptive cancer immunotherapy. High cytotoxicity of NK-92 has previously been shown against malignant cells of hematologic origin in preclinical studies, and general safety of infusion of NK-92 cells has been established in phase I clinical trials. To enhance their therapeutic utility, we genetically modified NK-92 cells to express chimeric antigen receptors (CAR) specific for tumor-associated surface antigens. Such CAR were composed of a tumor-specific scFv antibody fragment fused via hinge and transmembrane domains to intracellular signaling moieties such as CD3 zeta chain, or composite fusion molecules also containing a costimulatory protein domain in addition to CD3 zeta. For development towards clinical applications, here a codon-optimized second generation CAR was constructed that consists of an ErbB2-specific scFv antibody domain fused via a linker to a composite CD28-CD3 zeta signaling domain. GMP-compliant protocols for vector production, lentiviral transduction and expansion of a genetically modified NK-92 single cell clone (NK-92/5.28.z) were established. Functional analysis of NK-92/5.28.z cells revealed high and stable CAR expression, selective cytotoxicity against ErbB2-expressing but otherwise NK-resistant tumor cells of different origins in vitro, as well as homing to ErbB2-expressing tumors in vivo. Furthermore, antigen specificity and selective cytotoxicity of these cells were retained in vivo, resulting in antitumoral activity against subcutaneous and intracranial glioblastoma xenografts in NSG mice. Ongoing work now focuses on the development of these cells for adoptive immunotherapy of ErbB2-positive glioblastoma.

